# Nutritional characteristics of marine fish *Sardinella zunasi* Bleeker and immunostimulatory activities of its glycoprotein

**DOI:** 10.1039/c9ra04913d

**Published:** 2019-09-24

**Authors:** Yu Han, Huili Hao, Lihong Yang, Guolian Chen, Yucong Wen, Riming Huang

**Affiliations:** Guangdong Provincial Key Laboratory of Food Quality and Safety, College of Food Science, South China Agricultural University Guangzhou 510642 China hanyu@stu.scau.edu.cn hhlhaohuili@stu.scau.edu.cn guolianchen@scau.edu.cn vtchong@hotmail.com huangriming@scau.edu.cn +86 20 8528 3448; Shenzhen Shajing People's Hospital, Guangzhou University of Chinese Medicine Shenzhen China 13570253038@163.com

## Abstract

*Sardinella zunasi* Bleeker, an edible and medicinal marine fish, is largely distributed in tropical oceans. However, the chemical composition and nutritional properties of this species have not yet been investigated. In the present study, proximate composition, fatty acids, amino acids, taurine, and minerals of *S. zunasi* Bleeker were characterized, and the immunostimulatory properties of its glycoprotein were evaluated. The results indicated the presence of crude protein (19.66%), crude lipid (6.29%) and carbohydrate (0.74%) in *S. zunasi* Bleeker; monounsaturated fatty acids and polyunsaturated fatty acids in the fatty acid composition of *S. zunasi* Bleeker were 25.00% and 31.01%, respectively; *S. zunasi* Bleeker was rich in taurine (219 mg/100 g) and essential amino acids (5.57 g/100 g). In addition, the glycoprotein of *S. zunasi* consisted of protein and sugars, with a total content of 34.25% and 16.27%, respectively. The glycoprotein showed significant effects on promoting NO, TNF-α and IL-6 in a dose-dependent manner in RAW264.7 macrophage cells. Thus, these findings provide a scientific basis for the further utilization of glycoprotein from *S. zunasi* Bleeker.

## Abstract 2

Glycoprotein from the dible and medicinal marine fish *Sardinella zunasi* Bleeker possessed significant immunostimulatory activities in RAW264.7 macrophage cells.

## Graphical abstract

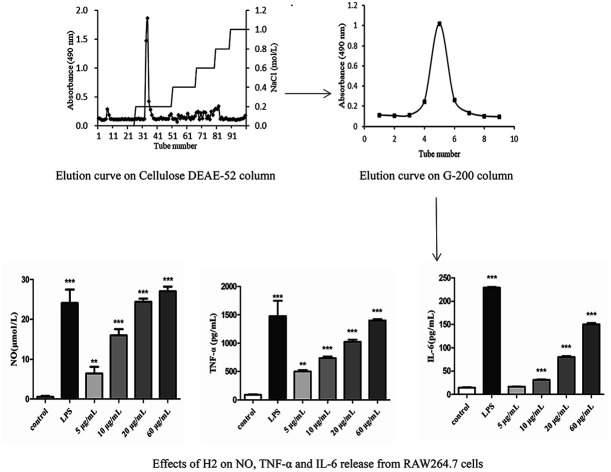

## 1. Introduction

Fish is a healthy food with numerous benefits to human health. It has been consumed in large quantities not only because it is a source of high nutritional quality protein, but also as a significant reserve of polyunsaturated fatty acids.^1^ Fish muscle is more digestible than other animal protein due to its lower level of connective tissue. Fish fat is one of the few natural food sources of vitamin D and contains important amounts of vitamins A and E.^2^ Fish is a perfect supplement to a high cereal diet because of its high lysine content.^3^ There is plenty of evidence that the consumption of fish reduces the risk of coronary heart disease.^4–7^ Marine fish is a rich source of high-quality protein, lipids, as well as all kinds of vitamins and minerals. It has been reported that the long-chain polyunsaturated (omega-3) fatty acids rich in marine fish, and these polyunsaturated fatty acids can reduce various pathophysiologic abnormalities including anoxic ventricular arrhythmia, atherogenesis, hypertension, blood clotting and cardiovascular disease.^8,9^

*S. zunasi* Bleeker, an edible and medicinal marine fish, belonging to genus *Sardinella*, family Clupeidae, is widely distributed in the coastal areas of tropical oceans, such as the Philippines, Japan, Korea and China. The species of genus *Sardinella* are taken as a good source of bioactive components. Previous investigation of genus *Sardinella* species has exhibited that the chemical or nutritional composition is rich in proteins, lipids (especially polyunsaturated fatty acids), and minerals^10–13^ and significant biological properties such as antioxidant,^14,15^ antibacterial,^16^ anticancer,^17^ hepatoprotective and nephroprotective,^18^ hypolipidemic, antiobesity and cardioprotective effects.^19^ However, there is still a lack of more data about the chemical or nutritional composition of genus *Sardinella*, and there is no report about the chemical or nutritional composition of *S. zunasi* Bleeker. Thus, it's necessary to investigate the chemical and nutritional composition of *S. zunasi* Bleeker before any in-depth study.

In order to make more effective utilization of *S. zunasi* Bleeker, the present work aims to determine the chemical and nutritional components including crude protein, crude fat, total carbohydrates, amino acids, taurine, fatty acids and minerals. In addition, the immunomodulatory effect of its glycoprotein on the murine RAW264.7 macrophage cell lines, including evaluating the effects on the proliferation of RAW264.7 cells, phagocytic uptake, the production of nitric oxide (NO), TNF-α, and IL-6 on RAW264.7 cells, was investigated. The results of this work might provide a useful information for further investigation of biological components from *S. zunasi* Bleeker and their utilization.

## 2. Materials and methods

### 2.1. Specimens of fish

*S. zunasi* Bleeker was purchased from Zhanjiang, Guangdong Province, China in August 2017. A voucher specimen (no. 20170803) was deposited in Guangdong Provincial Key Laboratory of Food and Safety, South China Agricultural University, China. After collection, fresh fish was packed in plastic bags, preserved in ice and taken immediately to the laboratory, it was preserved in a −20 °C refrigerator for about half a month before further treatments. The whole fish were thoroughly washed with running water, cut into pieces and then homogenised in a mincer before the analysis of nutritional characteristics and preparation of crude glycoprotein (H1) and pure glycoprotein (H2). Approximately 20 kilograms of fish was used for the entire study.

### 2.2. Standards and reagents

Standard mixtures of fatty acid methyl ester were purchased from Nu-Chek Prep (Inc., Elysian, MN, USA). All sugars (fructose, rhamnose, arabinose, xylose, mannose, glucose and galactose) were purchased from Aladdin Industrial Corporation (Shanghai, China). Murine RAW264.7 macrophage cells were obtained from Jinan University (Guangzhou, China). Lipopolysaccharides (LPS) and 3-(4,5-dimethyl-2-thiazolyl)-2,5-diphenyl-2-*H*-tetrazolium bromide (MTT) were purchased from Sigma Co. (Mo, U.S.A.). Dulbecco Modified Eagle Medium (DMEM), fetal bovine serum (FBS), and penicillin–streptomycin were purchased from Gibco Life Technologies (Grand Island, NY.). Nitric oxide (NO) kit was purchased from Beyotime Biotechnology Co. Ltd. Mouse TNF-α, and IL-6 ELISA kits were purchased from NeoBioscience Biotechnology Co. Ltd. DEAE-Cellulose 52 and Sephadex G-200 were purchased from Shanghai Yuanye Bio-Technology Co. Ltd. The other chemicals used in this research were of analytical grade.

### 2.3. Proximate composition analysis

The fish was washed and removed the surface water on the skin before composition analysis. The moisture, crude protein, crude lipid, and ash contents were analysed according to AOAC official methods.^20^ Moisture content was determined using a hot-air oven at 105 °C for 6 h until constant weight was reached. Nitrogen content was measured by the Kjeldahl method adapted from where protein content is estimated by multiplying the nitrogen content by 6.25. Crude lipids were determined by Soxhlet extraction with petroleum ether as the solvent. Ash content was measured by heating the fish in a muffle furnace at 550 °C until the resultant ash was light grey in color. Carbohydrate content was determined using the phenol-sulfuric acid method.^21^

### 2.4. Nutritional composition analysis

#### 2.4.1. Fatty acid profile analysis

The fatty acid profile was obtained using normalization method. 5.0 g of sample was transferred to 250 mL radius flask and mixed with 100 mg of pyrogallic acid, 2.0 mL of 95% ethanol, 4.0 mL of deionized water, several zeolites and 10 mL of 8.3 M HCl. The mixture was hydrolysed in a 75 °C water bath for 40 min and shook every 10 min. 10 mL of 95% ethanol was added to the hydrolysate after cooling and then transferred to separating funnel. The radius flask was washed with 50 mL of diethyl ether and petroleum ether (1 : 1) mixed solution and the flushing fluid also transferred to separating funnel. After shaking for 5 min, the ether layer was collected, and then evaporated to dryness. 8 mL of 2% sodium hydroxide methanol solution and 7 mL of 15% boron trifluoride methanol solution were added to the extraction during heating in an 80 °C water bath for 5 min, and then cooled to room temperature quickly. 20 mL of *n*-heptane was added, after 2 min shaking, saturated sodium chloride solution was added. After stratification, 5 mL of *n*-heptane was taken from the top to a tube and shook with 4 g of anhydrous sodium sulfate for 1 min, the upper solution was taken for further analysis.

Fatty acids were determined by a gas chromatography 7820A (Agilent Technologies, USA), equipped with a flame ionization detector (FID) and a HP88 capillary column (100 m length × 0.25 mm ID × 0.2 μm film thickness). Purified nitrogen was the carrier gas at a flow rate of 1 mL min^−1^. Sample of 1.0 μL was injected with a split ratio of 100 : 1. The injector and detector temperature were 270 °C and 280 °C, respectively. The column temperature was set at 100 °C for 13 min, followed by a 10 °C min^−1^ heating ramp to 180 °C, which was held for 6 min. Then the temperature was increased to 200 °C at a rate of 1 °C min^−1^ and held for 20 min. Finally, it was increased to 230 °C at a rate of 4 °C min^−1^ and held for 10.5 min. Fatty acids were identified by comparison of retention time with standard mixtures of fatty acid methyl ester, the composition of fatty acids was expressed in relative percentage of total fatty acids according to their peak areas.^22^

#### 2.4.2. Amino acid profile analysis

Approximately 1 g sample was put into a vial and hydrolysed with 10 mL of 6 M hydrochloric acid at 110 °C under a nitrogen atmosphere for 24 h. The received hydrolysed evaporated to dryness in the flow of warm water. The solid residual was dissolved in 1 mL of sodium citrate buffers (0.2 M) with pH 2.2 and filtered through a 0.22 μm filter before injected into the amino acid analyzer S-433D (Sykam, Germany). For tryptophan analysis, 1 g sample was digested with 4 M NaOH at 110 °C for 20 h under nitrogen gas. The hydrolysate was then neutralized to pH 7.0 with 6 M HCl and added sodium citrate buffers to reach the constant volume and filtered through a 0.22 μm filter before injected into an Agilent 1260 (Agilent Technologies, USA) high performance liquid chromatography (HPLC) system. The amino acid results are expressed in mg of amino acids per g protein.

#### 2.4.3. Taurine analysis

The taurine content was determined by an Agilent 1260 (Agilent Technologies, USA) HPLC system. 5 g sample was put into a conical flask, 20 mL of deionized water was added and sonicated at 40 °C for 10 min. Then the solution was mixed with 50 mL of 30 g L^−1^ metaphosphoric acid under ultrasonic treatment for another 10 min. The mixture was transferred to volumetric flask and diluted with distilled water to 100 mL. The sample was centrifuged at 5000 rpm for 10 min. The concentrated supernatant was filtered through an ash-free filter and 20 μL of them was injected into the HPLC equipped with sodium ion column (25 cm × 4.6 mm). The excitation and emission wavelengths were 340 nm and 450 nm, respectively. The mobile phase consisted of trisodium citrate solution which delivered to the column at a flow rate of 0.4 mL min^−1^. The result was expressed in mg of taurine content per 100 g fish meat wet weight.

#### 2.4.4. Mineral elements analysis

The mineral elements were measured by inductively coupled plasma optical emission spectrometry 725-ES (Agilent Technologies, USA). 1 g sample was digested with 15 mL of HNO_3_ for 30 min using microwave digestion system. 2 mL deionized water was added after cooling and continue heating till evaporated to nearly dry. After that, it was diluted with deionized water to 50 mL. The solution was filtered through an ash-free filter before analyzed. A blank digest was also carried out in the same way.

### 2.5. Glycoproteins extraction and purification

The extraction and purification of glycoprotein from *S. zunasi* Bleeker were carried out using the methods described previously.^23^ In brief, the fish was cut into pieces and ground up in a blender, then extracted twice by using a hot water method with the conditions of liquid/solid ratio of 20 : 1, temperature of 95 °C and extraction time of 2 h. The combined aqueous extracts were concentrated in a rotary evaporator under reduced pressure at 50 °C and then filtered. The free proteins in the extracts were removed by using Sevag reagent (chloroform/*n*-butanol, v/v = 4 : 1). The deproteinized solution was precipitated with addition of ethanol to reach a final ethanol concentration of 75% at 4 °C for 24 h. Following centrifugation at 12 000 rpm for 15 min, the precipitates were washed sequentially with anhydrous ethanol and acetone, and then dialyzed against deionized water for 2 days and lyophilized as crude glycoprotein (H1).

The crude glycoprotein (H1) was dissolved in distilled water and separated by a DEAE-Cellulose 52 column (2.0 cm × 40 cm). The column was eluted with distilled water and a stepwise NaCl gradient (0–1 M). One independent elution peak of the glycoprotein was obtained by phenol-sulfuric acid method at 490 nm. The glycoprotein fraction was collected, dialyzed, concentrated, and further loaded into Sephadex G-200 column (1.8 cm × 50 cm) with distilled water at a flow rate of 0.5 mL min^−1^. One independent elution peak of the glycoprotein was obtained by phenol-sulfuric acid method at 490 nm. One main fraction was finally obtained, dialyzed, lyophilized and denoted as H2.

### 2.6. Physicochemical characterization of H2

#### 2.6.1. Molecular weight analysis

The molecular weights of H2 was measured by a high-performance gel-permeation chromatography (HPGPC) on a Waters 1525 HPLC system fitted with two connecting columns, TSK G-5000PWXL (7.8 mm × 300 mm) and TSK G-3000PWXL (7.8 mm × 300 mm), and monitored with a Waters 2414 differential refractive index detector. 10 μL of 2 mg mL^−1^ H2 was injected to the system and eluted with 0.02 M KH_2_PO_4_ at a flow rate of 0.6 mL min^−1^ at 35 °C. The GPC system was calibrated before sample analysis with dextran standards (MW: 668 kDa, 410 kDa, 273 kDa, 148 kDa, 48.6 kDa, 23.8 kDa, 11.6 kDa, and 5.2 kDa).

#### 2.6.2. Monosaccharide analysis

The monosaccharide composition was analyzed by a Trace 1310 gas chromatography (GC) coupled with TSQ 8000 Evo mass spectrometry (MS) (Thermo Fisher Scientific, USA). 10 mg sample was hydrolyzed with 4 mL of 2 M trifluoroacetic acid (TFA) at 130 °C in a sealed glass tube for 4 h. 2 mL of methanol was used to remove excess TFA in a rotary evaporator under reduced pressure at 50 °C for three times after completing the hydrolysis. Then 10 mg of hydroxylamine hydrochloride and 2 mL of pyridine were added to the tube and incubated at 90 °C for 40 min. After that, 2 mL of acetic anhydride was added and incubated at 90 °C for another 40 min. 2 mL deionized water was added to eliminate the reaction. Acetylated derivatives were extracted with 3 mL chloroform for three times, and chloroform fractions were collected and concentrated at 40 °C. The residuum was dissolved with chloroform and 1 μL of it was injected to the system. Analytes were separated in the GC with a TG-5MS GC column (60 m length × 0.25 mm ID × 0.5 μm film thickness) at 1.2 mL min^−1^ helium flow and detected by MS using full scan mode (*m*/*z* 50–550). The injector and detector temperature were 230 °C and 280 °C, respectively. The column temperature was programmed as follows: 110 °C for 1 min; a 15 °C min^−1^ heating ramp to 180 °C, which was held for 1 min; a heating ramp to 280 °C at a rate of 2.5 °C min^−1^ and held for 10 min. The standard monosaccharides were prepared and subjected to the system in the same way.

#### 2.6.3. FTIR spectroscopy

The FT-IR spectrum for H2 was determined using a Fourier Transform Infrared (FT-IR) in the spectrophotometer with a frequency range of 4000–400 cm^−1^. H2 (1–2 mg) was adequately dried and ground with spectroscopic grade KBr powder (100 mg), then pressed into a 1 mm pellet for FT-IR measurement using a VERTEX 70 FT-IR infrared spectrometer (Bruker, Germany).

### 2.7. Immunomodulatory activity of H2

#### 2.7.1. Cell culture

Murine RAW264.7 macrophage cells were cultured in DMEM medium supplemented with 10% (v/v) FBS and 1% (v/v) penicillin–streptomycin at 37 °C in a humidified 5% CO_2_ atmosphere.

#### 2.7.2. Cell viability assay

Cell viability was determined by MTT method.^24,25^ RAW264.7 macrophage cells were seeded in 96-well plates at a density of 3 × 10^4^ cells mL^−1^ in 100 μL culture medium at an incubator (37 °C, 5% CO_2_). After incubation for 24 h, the cells were treated with 100 μL of different concentrations of H2 (10, 20, 40, 60, 80, 100, 120 and 140 μg mL^−1^) and incubated for another 24 h. In addition, equivalent volume of culture medium was used as the blank control. After treatment, 10 μL of MTT at 5 mg mL^−1^ was added to each well and further incubation for 4 h. At last, cell supernatant was moved and 100 μL of dimethyl sulfoxide (DMSO) were added. After shaking for 10 min, absorbance then was recorded at 540 nm wavelength using an automated microplate reader SpectraMax i3x (Molecular Devices, Austria). The cell survival ratio was expressed as a percentage of the control using the following formula:Cell viability (%) = (*A*_2_ − *A*_0_)/(*A*_1_ − *A*_0_) × 100where *A*_1_ was the absorbance of the control group; *A*_2_ was the absorbance of the test samples group; *A*_0_ was the absorbance of group treated with only culture medium.

#### 2.7.3. Phagocytic assay

Effects of H2 on the phagocytosis of RAW264.7 cells were measured by neutral red uptake method.^26^ RAW264.7 macrophage cells were seeded in 96-well plates at a density of 3 × 10^4^ cells mL^−1^ in 100 μL culture medium at an incubator (37 °C, 5% CO_2_). After cells were cultured with 100 μL of various concentrations (10, 20, 40, 60, 80, 100 and 120 μg mL^−1^) of H2 or LPS (2 μg mL^−1^) for 24 h, the culture medium was removed and 100 μL per well 0.1% neutral red solution was added. The plates were incubated for 1 h and cells were washed with 0.01 M PBS (pH 7.4) three times to remove excess neutral red. 100 μL per well lysis solutions (ethanol/acetic acid 1 : 1) were added, and then the cells were lysis for 30 min at room temperature. The absorbance was determined at 540 nm by microplate reader (Molecular Devices, Austria). The phagocytic rate was calculated by the following formula:Phagocytosis rate (%) = (*A*_2_ − *A*_0_)/(*A*_1_ − *A*_0_) × 100where *A*_1_ was the absorbance of the control group; *A*_2_ was the absorbance of the test samples group; *A*_0_ was the absorbance of group treated with only culture medium.

#### 2.7.4. Nitric oxide (NO) assay

NO levels in the cultured supernatants were measured by the Griess regent as described previously.^27^ RAW264.7 macrophage cells were seeded in 24-well plates at a density of 5 × 10^5^ cells mL^−1^ in 500 μL culture medium at an incubator (37 °C, 5% CO_2_). After incubation for 18 h, the cells were treated with 1000 μL of LPS (2 μg mL^−1^), a series of concentrations of H2 (5, 10, 20 and 60 μg mL^−1^), respectively, and incubated for 24 h. Then Griess reagent (50 μL) was added to 50 μL of collected supernatant and absorbance at 540 nm wavelength was recorded. Griess method is based on the determination of nitrite ions obtained by quantitative reduction (greater than 90%) of nitrate ions present in the sample. Therefore, the sum of nitrite and nitrate ion concentrations is measured. Nitrate concentration is deduced by subtracting the original nitrite from sample of the total nitrite concentration (sum of these NO_2_ with reduced NO_3_). A standard solution of NaNO_3_ was used for calibration.

#### 2.7.5. TNF-α and IL-6 determination

RAW264.7 macrophage cells were incubated in 24-well plates at a density of 5 × 10^5^ cells mL^−1^ in 500 μL culture medium at an incubator (37 °C, 5% CO_2_). After incubation for 18 h, the cells were treated with 1000 μL of a series of concentrations of H2 (5, 10, 20 and 60 μg mL^−1^), respectively, and incubated for 24 h. Cells with culture medium were used as a negative control and LPS (2 μg mL^−1^) was used as a positive control. Cytokines IL-6 and TNF-α levels were assessed using an immune enzymatic assay (ELISA) kit according to the manufacturer instructions. Samples were analyzed in triplicate and optical density was determined at 450 nm.

### 2.8. Statistical analysis

Data were expressed as mean ± SD, at least 3 independent experiments for each sample except fatty acids analysis of *S. zunasi* Bleeker and composition analysis and amino acid composition of H2. Statistical significance was calculated by one-way analysis of variance ANOVA followed by Turkey's test to determine the difference between groups (GraphPad Prism 5.0). Values of *P* < 0.05 were considered as statistically significant.

## 3. Results and discussion

### 3.1. Proximate composition

The proximate composition of *S. zunasi* Bleeker is presented in Table 1. Moisture (68.75% ± 0.79%) was the most abundant component in *S. zunasi* Bleeker, similar to *Pomatomus saltatrix* (70.87% ± 0.59%) and *Engraulis encrasicolus* (66.95% ± 0.64%).^28^ The protein content^29^ ranged from 17 g/100 g to 20 g/100 g for freshwater and 18 g/100 g to 22 g/100 g for marine fish. Crude protein content of *S. zunasi* Bleeker was 19.66% ± 0.15%, higher than that of *Mullus barbatus* (14.54% ± 0.00%), *Pomatomus saltatrix* (15.24% ± 0.55%) and *Scorpaena porcus* (16.91% ± 0.04%).^28^ Fishes were often classified as lean fish (lipid content < 5%), medium fat fish (5–10%), and fatty fish (>10%) on the basis of their fat content.^30^ Based on this classification, *S. zunasi* Bleeker was medium fat fish with 6.29% ± 0.74% lipid content, was similar to values reported in muscle of other fish species, such as sardines and mackerel.^1^ The ash content (6.16% ± 0.28%) of *S. zunasi* Bleeker was much higher than those of *Pampus argenteus* (2.25% ± 0.62%) and *Harpadon nehereus* (0.93% ± 0.11%).^31^ The major carbohydrate composition in fish muscle is glycogen that is a polymer of glucose. A typical muscle in a live fish may contain between 0.1 and 1% glycogen.^32^ In our study, we found carbohydrate content of *S. zunasi* Bleeker was 0.74% ± 0.01%, which agreed with the value reported in literature.^32^

**Table 1:** The proximate composition of *S. zunasi* Bleeker (mean ± SD, *n* = 3)

Proximate composition	Content^a^ (%)
Moisture	68.75 ± 0.79
Crude protein	19.66 ± 0.15
Crude lipid	6.29 ± 0.74
Ash	6.16 ± 0.28
Carbohydrate	0.74 ± 0.01

a Values expressed as wet weight.

### 3.2. Nutritional composition analysis

#### 3.2.1. Fatty acid content

The fatty acid composition of *S. zunasi* Bleeker is presented in Table 2. Twenty-seven fatty acids composed of C12:0 to C24:1, were identified. Fatty acids were generally divided into three categories: saturated fatty acids (SFA), monounsaturated fatty acids (MUFA) and polyunsaturated fatty acids (PUFA). These three fatty acids were found in 43.67, 25.00 and 31.01 percent of *S. zunasi* Bleeker, respectively. In recent decades, the WHO has recommended reduced consumption of saturated fatty acids based on their effects in increasing low density lipoprotein cholesterol (LDL-c) and risks of heart diseases.^33^ The marine fish *S. zunasi* Bleeker contained more UFA than SFA, the PUFA/SFA ratio (0.71) was higher than 0.45 that is an essential value of high nutritional food. Besides, EPA (Eicosapntemacnioc Acid) and DHA (Docosahexaenoic Acid) were important source of n-3 PUFA, together accounting for 20.30% of total fatty acids in *S. zunasi* Bleeker, higher than those of white herring, small yellow croaker and spotted maigre,^22^ indicated *S. zunasi* Bleeker was a healthy source of fatty acids.

**Table 2:** The fatty acid composition of *S. zunasi* Bleeker

Fatty acids	Content^a^ (%)
C12:0	0.14
C13:0	0.16
C14:0	8.60
C14:1	0.11
C15:0	0.91
C16:0	21.8
C16:1	7.60
C17:0	1.40
C17:1	1.40
C18:0	8.50
C18:1	12.40
C18:2	3.10
C18:3	1.30
C18:4	0.75
C20:0	1.10
C20:1	1.50
C20:2	0.15
C20:3	0.21
C20:4 (ARA)	2.70
C20:5 (EPA)	9.60
C21:0	0.32
C22:0	0.50
C22:1	0.89
C22:5 (EPA)	2.50
C22:6 (DHA)	10.70
C24:0	0.24
C24:1	1.10
SFA^b^	43.67
MUFA^c^	25.00
PUFA^d^	31.01

a Values expressed as wet weight.

b SFA: saturated fatty acids.

c MUFA: monounsaturated fatty acids.

d PUFA: polyunsaturated fatty acids.

#### 3.2.2. Amino acids content

The content of amino acids (AAs) and the proportion of amino acid components determined the nutritional value of protein in food. Some amino acids, such as cysteine (or sulphur-containing amino acid), tyrosine (or aromatic amino acids), histidine and arginine are required by infants and growing children.^34^ Deficiency in these amino acids may hinder healing recovery process.^35^ AAs have been traditionally classified as nutritionally essential amino acids (EAA), nonessential amino acids (NEAA) or conditionally essential amino acids (CEAA).^36^ The amino acid profile of *S. zunasi* Bleeker is shown in Table 3. Seventeen amino acids were identified. All of the essential amino acids were found to be present in *S. zunasi* Bleeker, accounting for 36.41% of the total amino acid. The highest content was glutamic acid at 2.41 g/100 g, which agreed with the reported data of four commonly consumed marine fishes.^37^ Lysine (8.50%), leucine (7.39%) and valine (4.58%) were the three most abundant essential amino acids, while glutamine and glutamic acid (15.75%), aspartic acid (9.48%) and glycine (8.56%) constituted the three most abundant nonessential amino acids in *S. zunasi* Bleeker.

**Table 3:** The amino acid composition of *S. zunasi* Bleeker (mean ± SD, *n* = 3)

Amino acids	Content^a^ (g/100 g)	Percentage of TAA^c^ (%)
Asx (Asp + Asn)	1.45 ± 0.02	9.48
Thr^b^	0.68 ± 0.01	4.44
Ser	0.64 ± 0.01	4.18
Glx (Glu + Gln)	2.41 ± 0.02	15.75
Gly	1.31 ± 0.03	8.56
Ala	1.19 ± 0.02	7.77
Val^b^	0.70 ± 0.01	4.58
Met^b^	0.49 ± 0.01	3.20
Trp^b^	0.11 ± 0.00	0.72
Ile^b^	0.56 ± 0.01	3.66
Leu^b^	1.13 ± 0.01	7.39
Tyr	0.46 ± 0.01	3.00
Phe^b^	0.60 ± 0.00	3.92
His	0.37 ± 0.01	2.42
Lys^b^	1.30 ± 0.02	8.50
Arg	0.98 ± 0.01	6.40
Pro	0.92 ± 0.01	6.01
EAA	5.57	36.41
NEAA	9.73	63.59

a Values expressed as wet weight.

b Essential amino acids.

c TAA: total amino acids.

#### 3.2.3. Taurine content

Taurine, a derivative of cysteine, lacks a carboxyl group in structure and is not strictly an amino acid, but contains a sulfonic acid group, so it is called sulfonic acid. The content of taurine was measured as 219 mg/100 g (wet weight) in *S. zunasi* Bleeker, which was higher than that of other marine species, such as mackerel (78 mg/100 g) and albacore tuna (40 mg/100 g).^38^ Beneficial effects of taurine on cardiovascular risk factors have been proposed,^39–42^ and both a reduction in body weight,^43^ beneficial effects on blood lipids,^44^ anti-atherosclerotic, and anti-inflammatory effects have been observed.^40^ The anti-obesity effects of taurine may partly be due to suppression of inflammation in adipose tissue.^45^ Taurine supplementation has been found to increase adiponectin levels, and decrease markers of inflammation (high-sensitivity C-reactive protein).^46^ Thus, *S. zunasi* Bleeker could be a promising natural source of taurine with benefits for human health.

#### 3.2.4. Mineral contents

As shown in Table 4, eight minerals were quantified in *S. zunasi* Bleeker, including four macro minerals (Ca, K, Na, and Mg) and four trace minerals (Fe, Zn, Mn, and Cu). Ca (1240 ± 0.02 mg/100 g) was the major mineral in *S. zunasi* Bleeker, the content of which was found to be higher than that in *Sardinella longiceps* (523.9 ± 45.6 mg/100 g),^47^*Mullus surmeletus* (398.6 ± 105.9 mg kg^−1^) and *Upeneus moluccensis* (617.4 ± 90.5 mg kg^−1^),^48^ followed by K (308 ± 0.08 mg/100 g), Na (168 ± 0.04 mg/100 g) and Mg (56.30 ± 2.7 mg/100 g). Ca rich in *S. zunasi* Bleeker suggests that it could be a good choice for nutritional supplements. The analysis of microelements revealed that *S. zunasi* Bleeker possessed Fe (9.48 ± 0.05 mg/100 g), Zn (2.55 ± 0.06 mg/100 g) and Mn (0.59 ± 0.01 mg/100 g), and the content of Cu was less than 0.5 mg/100 g. Fe is one of the essential trace minerals that good for health, the level of Fe (9.48 ± 0.05 mg/100 g) in *S. zunasi* Bleeker was much higher than that in *Upeneus moluccensis* (7.3 mg kg^−1^) and *Mullus surmeletus* (6.4 mg kg^−1^),^48^ indicated *S. zunasi* Bleeker may be taken as a good supplement source of Fe with healthy benefits to human.^49^

**Table 4:** The elemental composition of *S. zunasi* Bleeker (mean ± SD, *n* = 3)

Elements	Content^a^ (mg/100 g)
Ca	1240 ± 0.02
K	308 ± 0.08
Na	168 ± 0.04
Mg	56.30 ± 2.7
Fe	9.48 ± 0.05
Zn	2.55 ± 0.06
Mn	0.59 ± 0.01
Cu	<0.5

a Values expressed as wet weight.

### 3.3. Extraction, purification, and physicochemical properties of H2

**Fig. 1:**
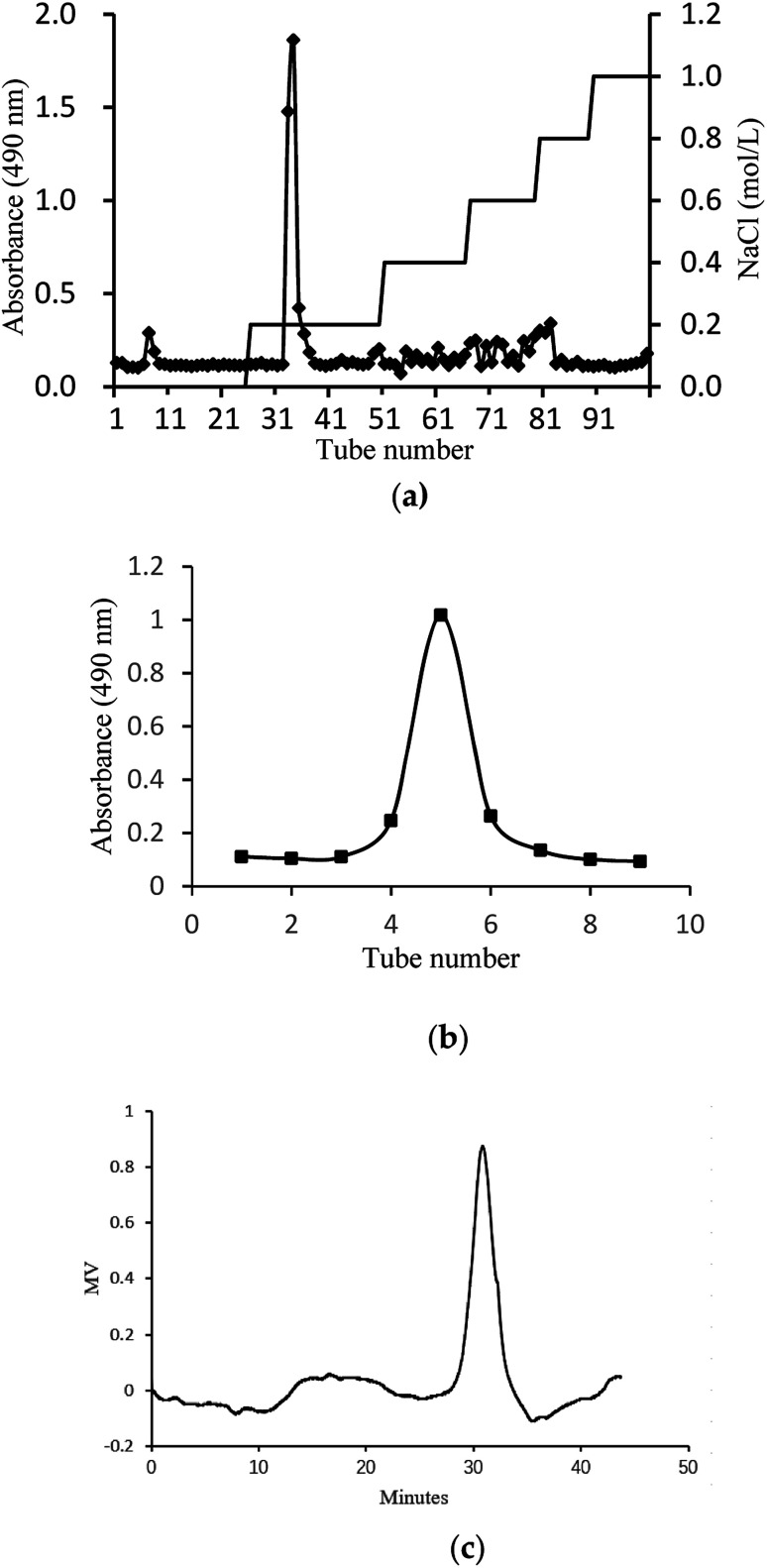
Preparation and physicochemical properties of H2. (a) Elution curve on Cellulose DEAE-52 column. (b) Elution curve on G-200 column and (c) HPGPC profile of H2.

In this study, crude glycoprotein (H1) was obtained from *S. zunasi* Bleeker by conventional hot water extraction, alcohol precipitation and deproteinization. Crude glycoprotein was further purified through anion-exchange chromatography according to differences in the existence of ionic groups. One fraction eluted with the 0.2 M sodium chloride solution was collected (Fig. 1a). After dialysis and concentration, it was further purified by Sephadex G-200 column, and then H2 was obtained (Fig. 1b). The physicochemical properties of H2 were shown in Table 5. The content of total sugar and protein were 16.27% and 34.25%, respectively. The main monosaccharide existed in H2 were mannose and galactose. The homogeneity and average molecular weight of H2 were measured by HPGPC shown in Fig. 1c. The GPC curves revealed that the fraction was represented by a single, sharp peak, indicated the glycoprotein was relatively homogeneous in molecular weight distribution. The average molecular weight of H2 was calculated as 7145 Da according to the equation of calibration curve.

**Table 5:** Results of composition analysis of H2

Total sugar content (%)	Protein content (%)	Sugar composition (molar ratio)	
		Mannose	Galactose
16.27	34.25	1.00	2.19

**Fig. 2:**
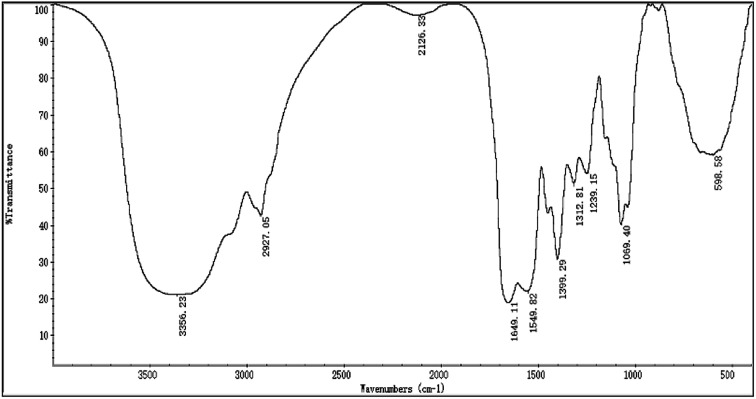
The FTIR spectra of H2.

The FTIR spectra of H2 is shown in Fig. 2, most of the absorption bands could be assigned according to data obtained in previous studies.^50–53^ The broad stretching intense peak at 3356 cm^−1^ indicated the presence of hydroxyl and amino groups,^54^ the 2927 cm^−1^ region corresponded to the C–H stretching vibration. The band at 1649 cm^−1^ was the C=O stretching vibration of amide group. The absorption peak at 1399 cm^−1^ represented the C–O–H carboxylic acid, and the strong peak observed at 1069 cm^−1^ was characteristic of all sugar derivatives and hydroxyl group.^55^ The spectra indicated H2 was a glycoprotein.

### 3.4. Immunomodulatory activity of H2

#### 3.4.1. Effects of H2 on RAW264.7 cells viability

**Fig. 3:**
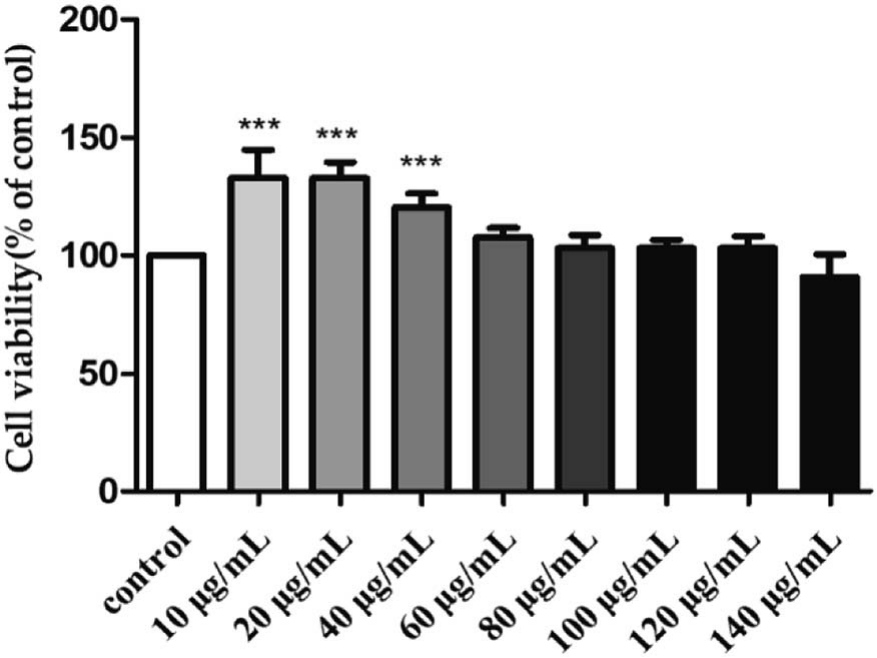
Effect of H2 on the viability of RAW264.7 cells. ***P < 0.001, *vs.* the control group.

To investigate the toxicity of H2 towards the RAW264.7 cells, their viability was evaluated by MTT assay after treatment with different H2 concentrations (10, 20, 40, 60, 80, 100, 120 and 140 μg mL^−1^) for 24 h. As shown in Fig. 3, with the rise of H2 concentrations from 10 μg mL^−1^ to 120 μg mL^−1^, the viability of RAW264.7 cells increased. At the concentrations of 10, 20 and 40 μg mL^−1^, the viability rates of RAW264.7 were 132.91 ± 11.85%, 132.71 ± 6.88% and 120.34 ± 5.95%, respectively. The viability was not significantly (*P* > 0.05) influenced by H2 at the concentrations from 60 μg mL^−1^ to 120 μg mL^−1^, compared with control group. When the concentration of H2 was up to 140 μg mL^−1^, the viability of RAW264.7 cells decreased. The results of present study indicated H2 at concentrations below 120 μg mL^−1^ was nontoxic to RAW264.7 cells, thus allowing concentrations from 10 μg mL^−1^ to 120 μg mL^−1^ being used for further study.

#### 3.4.2. Effects of H2 on phagocytic uptake

**Fig. 4:**
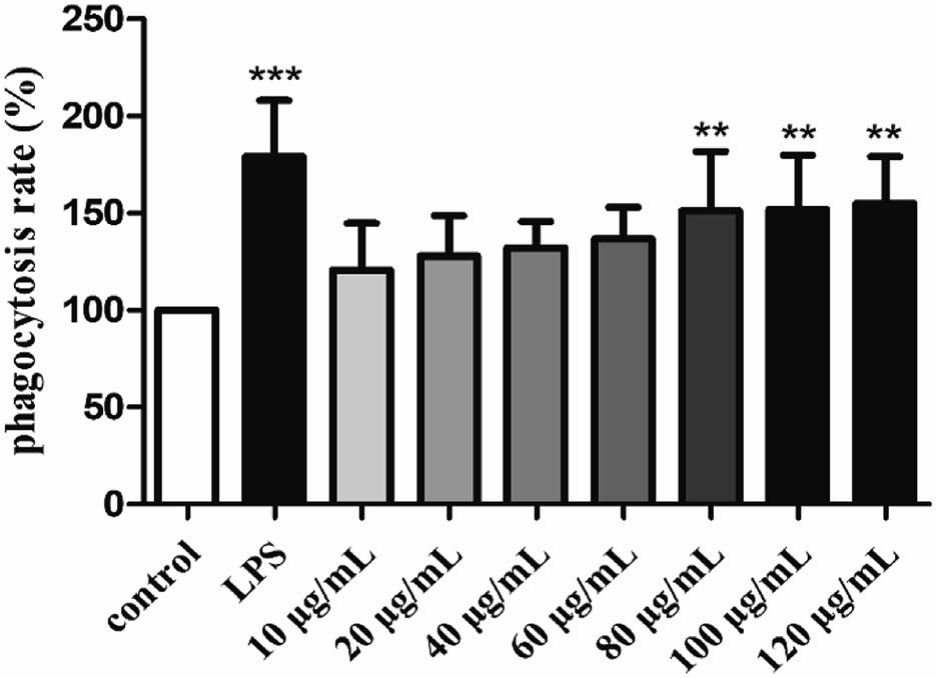
Effects of H2 on phagocytic uptake from RAW264.7 cells. **P < 0.01, ***P < 0.001, *vs.* the control group.

The phagocytic function of macrophages plays an important role in immune responses.^56^ Therefore, the effect of H2 on the phagocytic activities of RAW264.7 cells was examined by the uptake of neutral red. As showed in Fig. 4, H2 stimulated the phagocytic activity of RAW264.7 cells in a dose-dependent manner in the concentration ranged from 10 μg mL^−1^ to 120 μg mL^−1^ compared with the control group. The stimulatory effect of H2 on phagocytic activity of RAW264.7 cells did not differ significantly from that in the control group (*P* > 0.05) in the concentration ranged from 10 μg mL^−1^ to 60 μg mL^−1^. With the concentrations of H2 increased, the phagocytic activity was significant (*P* < 0.01) compared with the control group, indicated that H2 had abilities to promote phagocytic activities of macrophages at concentrations beyond 60 μg mL^−1^. However, the stimulatory effects of H2 on phagocytic activity were lower than that of LPS.

#### 3.4.3. Effects of H2 on NO production

**Fig. 5:**
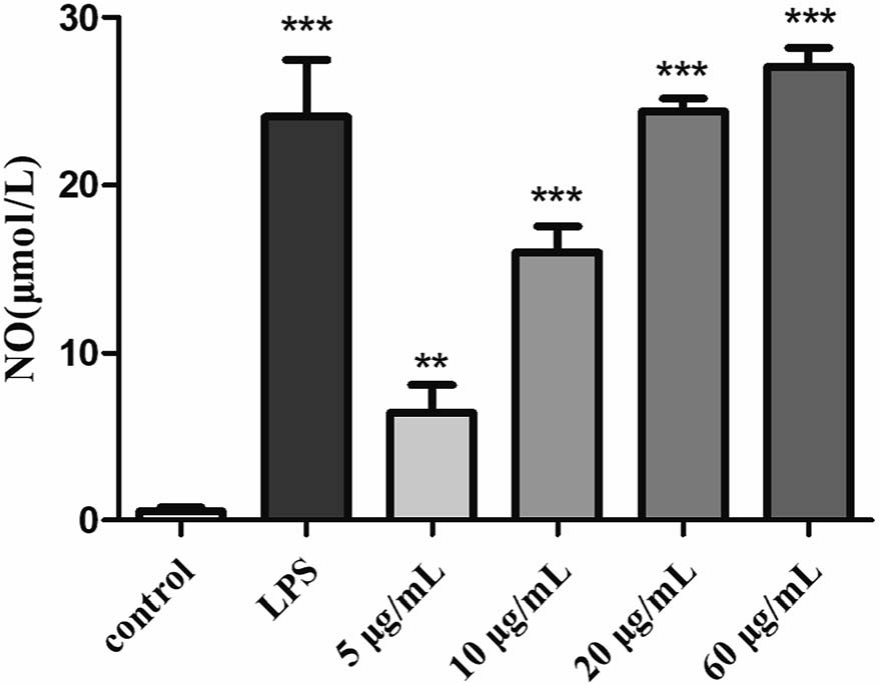
Effects of H2 on NO release from RAW264.7 cells. **P < 0.01, ***P < 0.001, *vs.* the control group.

As a kind of important signal transduction medium, NO plays an important role in the immune system.^57^ Analysis of the release of NO by activated macrophages can reflect the effects of *S. zunasi* Bleeker glycoprotein on immune function. Griess assay was used to determine the effect of H2 on the production of a macrophage-activating factor NO of RAW264.7 cells. The release of NO caused by H2 were shown in Fig. 5, the NO concentration of the culture supernatant of the RAW264.7 cells were increased by H2 in a dose-dependent manner from 5 μg mL^−1^ to 60 μg mL^−1^. Furthermore, the production of NO stimulated by a high concentration (60 μg mL^−1^) of H2 was even more than LPS-treated group.

#### 3.4.4. Effects of H2 on TNF-α and IL-6 production

**Fig. 6:**
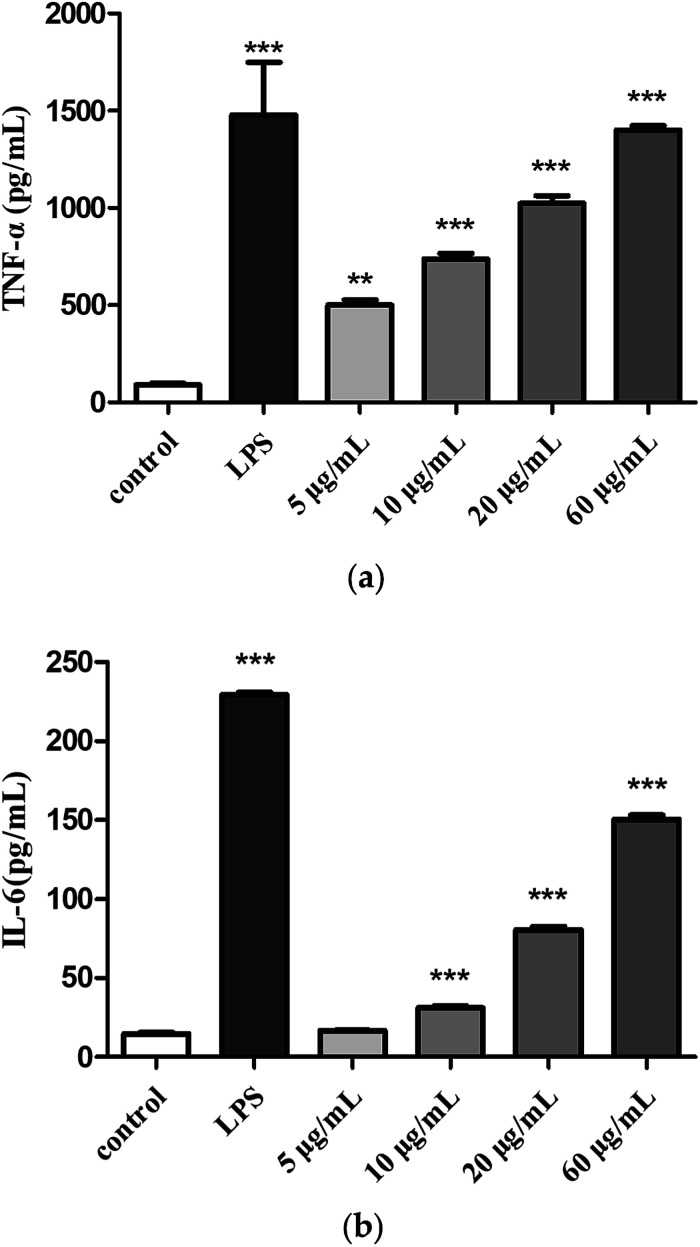
Effects of H2 on (a) TNF-α and (b) IL-6 from RAW264.7 cells. **P < 0.01, ***P < 0.001, *vs.* the control group.

Cytokines are small molecular proteins secreted by activated monocyte-macrophages and lymphocytes. TNF-α and IL-6 are two important cytokines that play a role in immune response. The production of TNF-α (Fig. 6a) and IL-6 (Fig. 6b) were low in the control group, compared with control group, treatment with H2 can induce TNF-α and IL-6 release in RAW264.7 cells in a dose-dependent manner from 5 μg mL^−1^ to 60 μg mL^−1^. The production of TNF-α (5, 10, 20 and 60 μg mL^−1^) was measured to be 5.5-fold, 8.1-fold, 15.5-fold and 16.9-fold of control group. Moreover, the production of TNF-α induced by H2 at concentration of 60 μg mL^−1^ showed a comparative level of the LPS-treated group. The production of IL-6 induced by low concentration (5 μg mL^−1^) of H2 showed no significance (*P* > 0.05) compared with control group, with the increase of H2 concentrations from 10 μg mL^−1^ to 60 μg mL^−1^, production of IL-6 increased, but still less than the LPS-treated group.

## 4. Conclusions

To sum up, the study revealed that *S. zunasi* Bleeker was a valuable marine fish for containing high level of protein and taurine. The amino acids and mineral composition indicated *S. zunasi* Bleeker was a good source of essential amino acids and micronutrients. Furthermore, *S. zunasi* Bleeker can be considered as a potential dietary source of DHA and EPA. The glycoprotein from *S. zunasi* Bleeker showed a significant immunostimulatory activity on RAW264.7 cells. It could not only promote cell proliferation and phagocytosis, but also promote inflammatory cytokines secretion. Thus, *S. zunasi* Bleeker can be considered as a nutritional supplement with a potential immunostimulatory property.

## Conflicts of interest

There are no conflicts to declare.

## Supplementary Material
